# But We Do Not Have a Critical Care Cardiologist? A Pragmatic Approach to Staffing the Cardiac Intensive Care Unit

**DOI:** 10.1016/j.jacadv.2025.101703

**Published:** 2025-04-16

**Authors:** Subaina Khalid, Julia Ma, Sarosh Janjua, Cynthia C. Taub, Courtney L. Maxey-Jones

**Affiliations:** a SUNY Upstate, Syracuse, New York, USA; b CNY Medical Services, PLLC, Baldwinsville, New York, USA

**Keywords:** cardiac critical care, cardiac intensive care unit, critical care cardiology

The cardiac intensive care unit (CICU) has grown from exclusively acute myocardial infarction care as a coronary care unit (CCU) to now encompass the care of the full spectrum of cardiology disorders causing critical illness. Historically, critical care medicine (CCM) physicians and cardiac subspecialists bore responsibility for the CCU/CICU. However, as cardiac critical care management has evolved in complexity, a perceived training gap has become more evident, and a demand for critical care cardiologists has developed. Unfortunately, a discrepancy exists between supply and demand. In a 2016 survey of 612 hospital systems with CICUs, 34.8% provided care through joint management between a general intensivist and cardiologist, 25.9% by a sole general intensivist, 19.3% by cardiologists, and only 8.5% by dual board-certified critical care cardiologists.^1^ Although joint management may provide for adequate care in the CICU, CCM staffing is already constrained. The reality is that many hospitals and entire regions, such as Syracuse, New York, do not have critical care cardiologists. We present a pragmatic approach to alternatives to CICU physician staffing.

## Background

Initially, there was no consensus on the optimal training model for preparing clinicians for careers in cardiac critical care, and 2 pathways were traditionally used:

1. Advanced Heart Failure and Transplant Cardiology (AHFTC) Training: Utilizing the overlap in cognitive and procedural abilities needed for cardiac critical care specialists, critical care ideas are incorporated into AHFTC training.^2^ Clinicians with AHFTC training are prepared to treat patients with severe heart failure and those who need sophisticated procedures like ventricular support devices and extracorporeal membrane oxygenation (ECMO).
2. CCM Training: Essential skills like ventilator management, multiorgan dysfunction management, and end-of-life care are taught during a year-long critical care training program. To manage CICU patients with complicated medical demands, it is becoming increasingly important for professionals to have broad critical care expertise.

Subsequently, it was determined that competency in managing contemporary CICU is optimized by dual certification in CCM and cardiovascular disease. Critical care abilities pertinent to CICU treatment, such as ventilator management, multiorgan failure, and end-of-life care, were emphasized in a recent study that polled dual-certified physicians.^3^ Crucially, only 17% believed that a general cardiology fellowship was sufficient to meet the requirements of patients in the present-day CICU. However, according to recent surveys, only 8.7% of hospitals employ dual-trained critical care cardiologists as unit chiefs, and only 14.7% of institutions have at least 1 critical care cardiology attending physician.^1^ Furthermore, a study of the Doximity physician database found that dual-certified physicians managed 0.47% of CICU admissions.^3^^,^^4^

Various models have been proposed to meet current demands for cardiac critical care. In 2001, the European Society of Cardiology (ECS) established a working group specifically for acute cardiovascular care, officially publishing recommendations regarding the structure, organization, and function of CICUs and intermediate cardiac units in 2005.^5^ Proposals for the structuring of CICUs were comprehensive; however, they did not specify training paradigms for CICU staffing, only stating that attending cardiologists should be able to manage “urgent cardiac situations, including rhythm, hemodynamic disturbances, and acute ischemia, and be adept with skills such as inserting an endotracheal tube, a temporary pacemaker, a catheter in the pulmonary artery, and a balloon in the aorta for counter-pulsation”.^5^ As the cardiovascular critical care field continued to progress, updated recommendations were published by the Acute Cardiovascular Care Association in 2016.^6^ These recommendations provide guidance for Acute Cardiovascular Care Association accreditation, advocating for at least 21 months of intensive care training, including 3 months of general intensive care and 6 months CICU as part of general cardiology training in addition to 1 year of subspecialty acute cardiovascular care training.^6^ The ECS also set competency standards with the design of an acute cardiovascular care certification program. Following the example set by the ECS, the American College of Cardiology Core Training Statement officially defined training pathways for cardiac critical care subspecialty training in 2015.^7^ Clinical competencies for specific tiers (level I to III) of training were established, with level III training representing the highest level of clinical competency, requiring dual cardiology and critical care board certification. Within level III training, 3 training models were proposed: 1) standard dual certification (36 months of cardiology and 6-12 months of clinical CCM training); 2) tailored dual certification (collaboration between CCM and cardiology, 3 years of cardiology, and 1 year of cardiac critical care training); and 3) unified 4-year training program in critical care cardiology. However, there remains little guidance regarding specific training competencies beyond these proposed training models.

## But what if we do not have a critical care cardiologist?

While the development of a pathway and training program for critical care-trained cardiologists will certainly advance the care of CICU patients, there remains a severe shortage of critical care cardiologists. Thus, we present a pragmatic and opinion-based comparison of alternative physicians to staff the CICU when critical care cardiologists are not available.

### Noninvasive cardiologists

Historically, the most common physician to staff a CICU is a noninvasive cardiologist. A cardiologist’s training is deeply rooted in diagnosing and managing all cardiovascular conditions, including coronary artery disease, heart failure, arrhythmias, and valvular disorders. Cardiologists, with their background in internal medicine and cardiology, are expert diagnosticians skilled in physical examination and interpreting diagnostic data, especially for the chronic conditions frequently seen in the CICU population. Cardiologists are proficient in interpreting key diagnostic tools such as EKGs, rhythm monitors, echocardiograms, cardiac CTs, MRIs, coronary angiograms, and right heart catheterizations. Given that the CICU population is aging and often sicker, cardiologists are central in coordinating multidisciplinary care, particularly for patients on mechanical circulatory support or durable left ventricular assist devices. Cardiologists' internal medicine background makes them well-suited to lead multidisciplinary teams in the CICU, working with surgeons, intensivists, and other specialists to provide comprehensive patient care. The limitation of having a noninvasive cardiologist staff the CICU is the need for support in the critical care aspects of care. Noninvasive cardiologists are unlikely to be experienced in the placement of invasive lines, ventilator management, or management of other critical organ dysfunction. The support of consulting CCM physicians can overcome this.

### Advanced heart failure and transplant cardiologists (AHFTC)

AHFTCs have an increased knowledge of complex cardiac disease with some overlap to critical care knowledge, making them a very suitable option for managing CICU patients. They are trained in advanced invasive monitoring, such as pulmonary artery catheter placement, and have a robust knowledge of temporary mechanical circulatory support (tMCS). Despite some critical care expertise, AHFTCs will likely require CCM support, particularly for airway management and mechanical ventilation assistance. The main limitation to increased CICU staffing by AHFTCs is the large deficit of need vs availability. Very few AHFTCs are available and are typically only found in large academic centers that offer durable left ventricular assist devices and heart transplantation.

### Interventional cardiologists

Another frequent physician used to staff CICUs is interventional cardiologists (ICs). Their expertise in managing cardiac emergencies, including emergent percutaneous coronary interventions, temporary pacemakers, pulmonary artery catheter insertions, tMCS device insertions, and ECMO cannulation, makes them a crucial part of the CICU team. Because these skills are in addition to their training as noninvasive cardiologists, ICs can make rapid, informed decisions about when and how to deploy advanced interventions. ICs may also require the support of consulting critical care physicians for some aspects of CICU care, albeit to a lesser extent than noninvasive cardiologists.

### Cardiac anesthesiologist intensivist

Cardiac anesthesiologist intensivists (CAIs) may be equivalent to cardiology critical care physicians, as CAIs are certified in critical care and have specialty knowledge in cardiac care. The unique skill set of a CAI could make them the best choice for managing patients in the CICU. CAIs traditionally staff the cardiovascular intensive care unit and CTICUs, caring for perioperative patients. CAIs are specifically trained in managing the intricate hemodynamics of critically ill patients, possess a deep knowledge of cardiovascular physiology and pharmacology, and are experts in peri-operative care. In the CICU, where patients often experience sudden cardiovascular collapse or require intensive monitoring and therapy, CAIs can leverage advanced techniques like invasive hemodynamic monitoring (eg, pulmonary artery catheter placement, temporary transvenous pacemaker placement, and transesophageal echocardiography). CAIs have extensive knowledge and experience in managing tMCS devices. CAIs often have more hands-on experience with these technologies than other physicians. Additionally, many CAIs have advanced beyond the management skills of tMCS to technical skills, including intraaortic balloon pump placement and removal, percutaneous ventricular assist devices placement and removal, and ECMO cannulation and decannulation. Importantly, CAIs are trained in critical illness beyond cardiac disease in the full spectrum of critical illness, including respiratory failure, renal failure, and other organ dysfunction. Their expertise in critical care allows them to address the complexities of multisystem failure, ensuring that every aspect of the patient's care is addressed. Another critical skill set of CAIs is their expertise in airway management and respiratory support, which allows for the optimization of care for the full cardiopulmonary system. Another consideration is that there is a shortage of CAIs, just as there is a shortage of critical care cardiologists and AHFTCs.

### CCM physicians

Another alternative physician to staff CICUs is CCM physicians, usually trained in pulmonary critical care. This option presents a trade-off of providing critical care support and the traditional knowledge and skill set of medical intensive care with the loss of cardiac-specific care. CCM physicians lack the foundational cardiology knowledge necessary to diagnose and treat advanced cardiac diagnoses. CCM physicians are well-trained across a variety of medical diagnoses. Still, the training and experience are typically in a traditional medical intensive care unit, which in hospitals with CICUs does not provide robust cardiac experience. CCM physicians are able to provide advanced ventilator management but may or may not provide airway management. They typically are comfortable with the placement of arterial lines and central lines but may not be able to provide advanced invasive lines such as pulmonary artery catheters and temporary transvenous pacers. Also, they do not have training or experience in tMCS or peri-operative care of cardiac patients. CCM physicians are an opportunity to advance the management of critical illness in the CICU; however, the benefit of receiving cardiac-specific care may be diminished in this model. If a CCM physician is the primary physician in the CICU, close support and partnership with a cardiology consult will be required.

### Cardiothoracic surgeons

Cardiothoracic surgeons are infrequently used physicians for CICU staffing, but they remain an option. At the conclusion of cardiothoracic surgery training, surgeons are qualified to care for critically ill patients. Cardiothoracic surgeons independently manage their postcardiac surgery patients in many cardiac surgery centers. These surgeons are highly experienced in dealing with the complications that arise after cardiac surgery, such as bleeding, arrhythmias, or graft failure. Although they may not have extensive expertise in heart failure management, their focus is on ensuring the postsurgical patient recovers from any complications that could affect cardiac function. In the absence of a critical care cardiologist, cardiothoracic surgeons are another option to staff the CICU, most commonly in a combined CICU/CTICU model. They may attend to the CICU patients and collaborate with other cardiologists and intensivists to manage the patient and help prevent further cardiac deterioration. Limitations are the shortage of cardiothoracic surgeons and the disadvantage in health care economics of a surgeon focusing time in the CICU rather than the operating room.

## Conclusions

CICU care has rapidly evolved, but a continued limitation of CICU development is the rarity of critical care cardiologists to manage the CICU. We have reviewed multiple alternatives to critical care cardiology staffing; however, many of the options remain limited in availability. While we work to address the shortage of critical care cardiologists, we cannot accept slowing the development of CICU care. Perhaps, in alignment with the rest of cardiology care, the CICU is presently best served by a heart team approach of multidisciplinary collaborative care.

## Funding support and author disclosures

Dr Maxey-Jones is an advisor/speaker for Atricure and J&J Medtech; an advisory board member/speaker for Haemonetics; an advisor/investor/owner of Doctorpedia; and an investor/owner of Romtech. All other authors have reported that they have no relationships relevant to the contents of this paper to disclose.
